# Nodular Fasciitis Mimicking Lymph Node Recurrence after Surgery for Sigmoid Colon Cancer: A Case Report

**DOI:** 10.70352/scrj.cr.25-0762

**Published:** 2026-04-17

**Authors:** Jumpei Ogawa, Yasuhiro Ishiyama, Yuto Arai, Kōhei Sekiguchi, Keisuke Yuki, Kazuharu Watanabe, Ichitarō Mochizuki, Yasuhito Suenaga, Yoshiaki Hara, Kazuhiro Narita, Manabu Amiki

**Affiliations:** 1 Department of Surgery, Kawasaki Saiwai Hospital, Kawasaki, Kanagawa, Japan; 2 Saitama Medical University International Medical Center, Hidaka, Saitama, Japan

**Keywords:** colorectal cancer, intra-abdominal lesion, nodular fasciitis

## Abstract

**INTRODUCTION:**

Nodular fasciitis (NF) is a benign, rapidly proliferating fibroblastic lesion that may show ^18^F-fluorodeoxyglucose (FDG) uptake on PET/CT, often mimicking malignancy. Postoperative intra-abdominal NF following colorectal cancer surgery is extremely rare.

**CASE PRESENTATION:**

A 56-year-old man underwent endoscopic mucosal resection for sigmoid colon cancer, followed by laparoscopic sigmoidectomy for submucosal invasive carcinoma (pT1b). Twelve months later, CT revealed a 13-mm mass near the superior rectal artery, and PET/CT showed mild FDG uptake (maximum standardized uptake value 1.13). Laparoscopic excision was performed, and histopathology confirmed NF. No recurrence has been observed after 3 years.

**CONCLUSIONS:**

Intra-abdominal NF may closely resemble lymph node recurrence after colorectal cancer surgery. Clinicians should consider NF in the differential diagnosis of postoperative intra-abdominal nodules with FDG uptake.

## Abbreviations

CA 19-9
: carbohydrate antigen 19-9
CEA
: carcinoembryonic antigen
FDG-PET
: ^18^F-fluorodeoxyglucose PET
NF
: nodular fasciitis
SUVmax
: maximum standardized uptake value

## INTRODUCTION

NF, first described by Konwaler et al.,^1)^ is a benign fibroblastic tumor that often presents as a rapidly enlarging soft tissue mass. It typically presents as a small (generally <2cm), rapidly growing mass arising in the subcutaneous tissue.^2)^ Cases of intra-abdominal NF are extremely rare, with only a limited number reported both domestically in Japan and internationally. NF may mimic malignancy due to rapid growth and FDG uptake.^3)^ Postoperative occurrence of NF following colorectal cancer surgery is extremely rare.^4)^ This report describes an intra-abdominal NF lesion that appeared similar to lymph node recurrence following sigmoid colon cancer surgery.

## CASE PRESENTATION

**Fig. 1:**
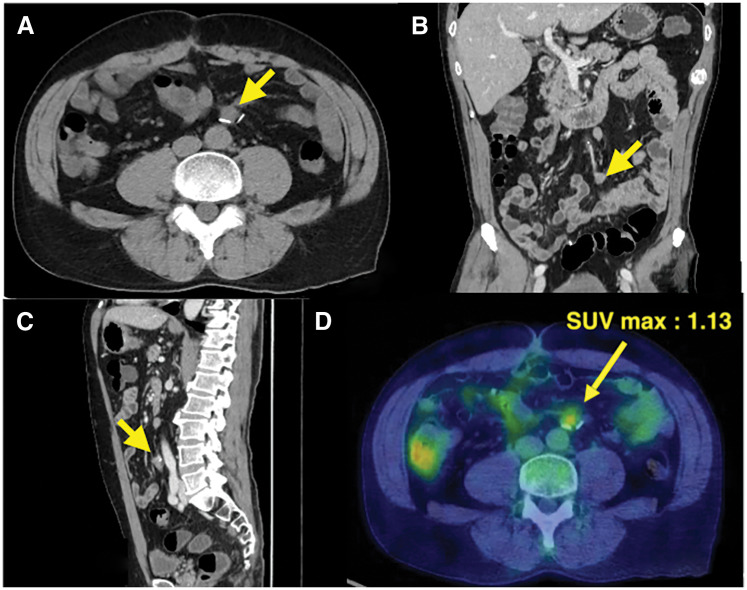
CT and FDG-PET images. (**A**–**C**) At 12 months postoperatively, CT images identified a 13-mm soft tissue lesion near the superior rectal artery stump (yellow arrow). (**D**) FDG-PET showed abnormal uptake at the stump of the superior rectal artery. A solitary lesion measuring 17 mm with a SUVmax of 1.13 was identified. FDG, ^18^F-fluorodeoxyglucose; SUVmax, maximum standardized uptake value

**Fig. 2:**
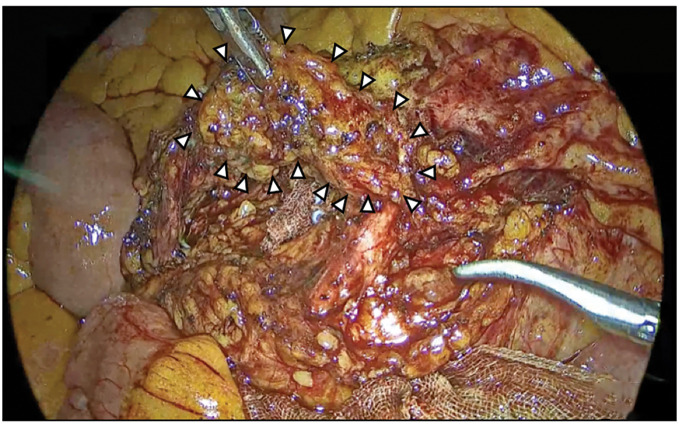
Intraoperative laparoscopic view showed a firm nodule around the stump of the superior rectal artery (arrowheads). Minimal adhesions were observed from the prior surgery.

**Fig. 3:**
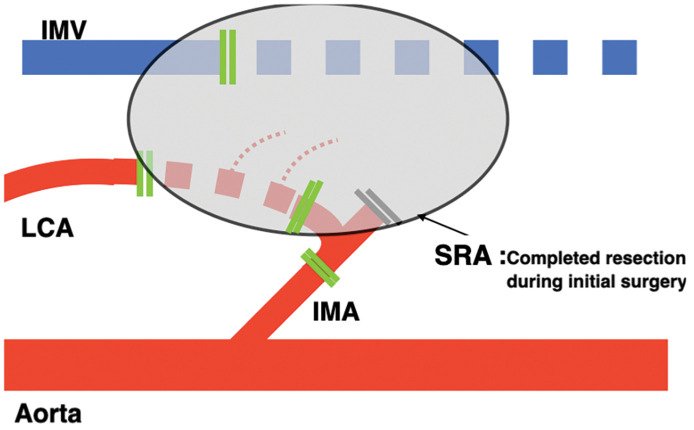
Schematic of en bloc resection of the tumor. Schematic showed involvement of the SRA, LCA, and a portion of the IMA vessels during laparoscopic surgery. IMA, inferior mesenteric artery; IMV, inferior mesenteric vein; LCA, left colic artery; SRA, superior rectal artery

A 56-year-old man presented after a positive fecal occult blood test. Colonoscopy revealed a 0-Isp lesion in the sigmoid colon that was removed via endoscopic mucosal resection. Pathology showed submucosal invasive carcinoma measuring 5000 μm (pT1b), thus prompting additional laparoscopic sigmoidectomy with D2 lymph node dissection. No residual carcinoma or lymph node metastasis was found, resulting in stage I (pT1bN0M0) disease. Postoperatively, follow-up was performed with blood tests every 3 months, including measurement of tumor markers CEA and CA19-9, and contrast-enhanced CT was obtained every 6 months for surveillance. Tumor marker levels remained within their normal ranges through 12 months after the initial surgery. No evidence of recurrence was detected on CT performed at 6 months postoperatively. However, at 12 months postoperatively, CT identified a 13-mm soft tissue lesion near the superior rectal artery stump (**Fig. 1A**–**1C**). FDG-PET/CT showed mild FDG uptake (SUVmax 1.13) (**Fig. 1D**). Lymph node recurrence was suspected, and laparoscopic resection was planned. Intraoperatively, a firm nodule surrounding the arterial stump was identified and excised en bloc with adjacent vascular structures (**Figs. 2** and **3**). The patient’s postoperative course was uneventful, and he was discharged on POD 4. No recurrence has been observed after 3 years, and tumor marker levels have remained within normal range throughout follow-up.

### Pathological findings

**Fig. 4:**
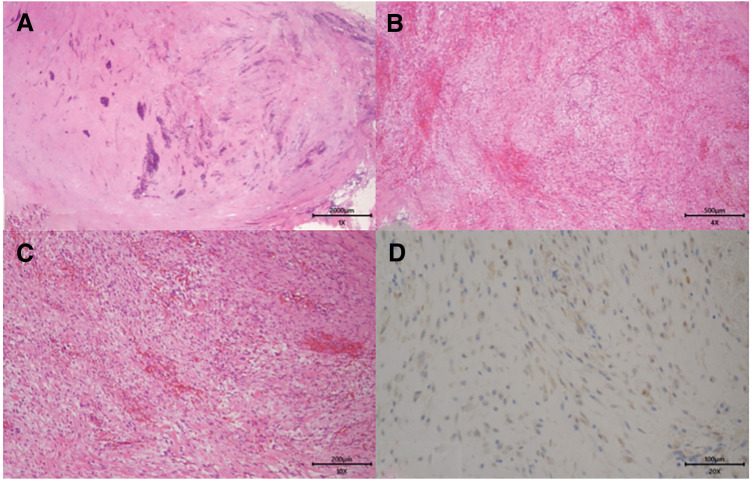
Histopathological findings of the resected specimen. Spindle-shaped fibroblasts arranged in fascicles with focal cellularity and interspersed mature collagen fibers were shown. Occasional foreign body-type giant cells were visible at the margins (**A**–**C**). β-Catenin staining showed cytoplasmic but not nuclear positivity (**D**).

The excised specimen included an 8-mm lymph node within a firm nodule. Histology showed spindle-shaped fibroblasts arranged in fascicles, focal hypercellularity, mature collagen deposition, and occasional foreign body-type giant cells (**Fig. 4A**–**4C**). β-catenin staining showed cytoplasmic but not nuclear positivity (**Fig. 4D**). In this case, no additional immunohistochemical staining or genetic testing was performed. These findings confirmed the diagnosis of NF.

## DISCUSSION

NF is a benign fibroblastic/myofibroblastic tumor as classified in the World Health Organization classification of soft tissue tumors. The diagnosis relies primarily on pathological findings and is made comprehensively based on histopathological features, immunohistochemical findings, genetic testing when available, and the clinical course.

NF may arise in multiple anatomical locations and affects both sexes equally. Typical sites include the upper extremities (48%), trunk, head and neck, and lower extremities.^5)^ Although mechanical stimulation has been suggested as a potential contributing factor,^1)^ many patients report no history of trauma.^4)^

Postoperative occurrence of NF following colorectal cancer surgery is exceptionally rare. A PubMed search using the keywords “nodular fasciitis” and “abdominal” from January 1985 to December 2025 identified 27 publications, of which only two reported cases of intra-abdominal involvement. Yamamoto et al. reported a postoperative case in which NF developed at a robotic surgery port site after rectal cancer resection.^4)^ Another case by Shiga et al. described mesenteric NF mimicking peritoneal carcinomatosis.^6)^ No prior reports, to our knowledge, have described intra-abdominal NF after sigmoid colon cancer surgery.

NF lesions are often smaller than 4 cm and may regress spontaneously, contributing to underdiagnosis. Stanley et al. noted spontaneous resolution in 9 of 11 cases within 8 weeks.^7)^ NF can resemble sarcoma due to rapid growth and cellularity and has been cited as the benign lesion most frequently misdiagnosed as sarcoma.^8)^ FDG uptake also overlaps with malignant tumors, with SUVmax values ranging from 1.9 to 11.9.^3)^

Molecular testing can serve as a useful diagnostic marker for NF. Erickson-Johnson et al. reported that approximately 92% of NF cases harbor rearrangements involving the *USP6* gene. *USP6* belongs to a large subfamily of deubiquitinating enzymes that are involved in various cellular processes, including intracellular transport, protein metabolism, inflammatory signaling, and cellular transformation. In most cases, *USP6* rearrangements involve *MYH9*–*USP6* gene fusions. Conversely, alternative fusion partners, such as *EIF5A* or *TPM4*, have been reported in cases with distinct clinicopathological features.^9,10)^

Histopathology and immunohistochemistry remain essential for a definitive diagnosis. Desmoid-type fibromatosis must be differentiated, as it usually shows nuclear β-catenin positivity due to *CTNNB1* mutations. In the present case, β-catenin staining was negative in nuclei, supporting the diagnosis of NF.

Leiomyosarcoma typically shows positive desmin immunostaining, which distinguishes it from NF.^11)^ The spindle cell pattern and stromal features observed in the present case are also consistent with previously described characteristics of NF.^12)^ Complete surgical excision is curative in most cases, with recurrence rates of around 1%.^13)^ Although the SUVmax value was low in this patient, malignancy could not be excluded without histology, thus necessitating surgical resection.

## CONCLUSIONS

Intra-abdominal NF is rare and may closely resemble lymph node recurrence after colorectal cancer surgery. NF should be considered in the differential diagnosis of postoperative intra-abdominal lesions with FDG uptake.

## References

[1] Konwaler BE, Keasbey L, Kaplan L. Subcutaneous pseudosarcomatous fibromatosis (fasciitis). Am J Clin Pathol 1955; 25: 241–52. 14361319 10.1093/ajcp/25.3.241

[2] Erickson-Johnson MR, Chou MM, Evers BR, et al. Nodular fasciitis: a novel model of transient neoplasia induced by *MYH9-USP6* gene fusion. Lab Invest 2011; 91: 1427–33. 21826056 10.1038/labinvest.2011.118

[3] Kubota K. From tumor biology to clinical PET: a review of positron emission tomography (PET) in oncology. Ann Nucl Med 2001; 15: 471–86. 11831394 10.1007/BF02988499

[4] Yamamoto A, Furuya S, Takiguchi K, et al. Nodular fasciitis growing at the port site of robotic surgery for rectal cancer. Surg Case Rep 2020; 6: 315. 33296059 10.1186/s40792-020-01049-8 PMC7726077

[5] Kim ST, Kim HJ, Park SW, et al. Nodular fasciitis in the head and neck: CT and MR imaging findings. AJNR Am J Neuroradiol 2005; 26: 2617–23. 16286411 PMC7976172

[6] Shiga M, Okamoto K, Matsumoto M, et al. Nodular fasciitis in the mesentery, a differential diagnosis of peritoneal carcinomatosis. World J Gastroenterol 2014; 20: 1361–4. 24574812 10.3748/wjg.v20.i5.1361 PMC3921520

[7] Stanley MW, Skoog L, Tani EM, et al. Nodular fasciitis: spontaneous resolution following diagnosis by fine-needle aspiration. Diagn Cytopathol 1993; 9: 322–4. 8519200 10.1002/dc.2840090316

[8] Weiss SW, Goldblum JR. Enzinger and Weiss’s soft tissue tumors, 5th ed. Philadelphia: Mosby Elsevier; 2007. p. 177–93.

[9] Rodriguez Pena MDC, Morlote D, Prieto Granada CN. Cutaneous nodular fasciitis with rare *TPM4-USP6* fusion. J Cutan Pathol 2022; 49: 196–9. 34643284 10.1111/cup.14151

[10] Lenz J, Michal M, Svajdler M, et al. Novel *EIF5A-USP6* gene fusion in nodular fasciitis associated with unusual pathologic features: a report of a case and review of the literature. Am J Dermatopathol 2020; 42: 539–43. 31880592 10.1097/DAD.0000000000001602

[11] Lazar AJ, Tuvin D, Hajibashi S, et al. Specific mutations in the β-*catenin* gene (*CTNNB*_*1*_) correlate with local recurrence in sporadic desmoid tumors. Am J Pathol 2008; 173: 1518–27. 18832571 10.2353/ajpath.2008.080475 PMC2570141

[12] Yan B, Li Y, Pan J, et al. Primary oral leiomyosarcoma: a retrospective clinical analysis of 20 cases. Oral Dis 2010; 16: 198–203. 20374505 10.1111/j.1601-0825.2009.01635.x

[13] Allen PW. Nodular fasciitis. Pathology 1972; 4: 9–26. 4501523 10.3109/00313027209068920

